# Appetite, Digestion and Health

**Published:** 1908-06

**Authors:** 


A Monthly Review of Medicine and Surgery.

*EDITOR:*

WILLIAM WARREN POTTER, M. D.

All communications, whether of a literary or business nature, books for review and exchanges, should be addressed to the editor 238 DELAWARE AVE., BUFFALO, N. Y

VOL. LXIII. JUNE, 1908. NO. 11

### Appetite, Digestion and Health

THE greatest assets a human being can possess, when health and longevity are considered, are appetite and good digestion. Speaking generally, the two go hand in hand though exceptionally they may fall apart. Not always, however, does the possessor of good appetite employ it discreetly. To minister appropriately to the demands of a healthy appetite is a rare gift and one that should be conserved with great judgment. Shakespere states the matter most concisely in those familiar words—"Now, good digestion wait on appetite and health on both."

It is not easy to lay down rules that shall apply to every individual nor, indeed, to even large groups of persons, because methods and habits of life are so variant; and because, further, occupation and individual peculiarity differ so widely. In other words, likes and dislikes, idiosyncrasies, and digestive capabilities enter largely into a solution of the questions involved in this topic. A few years ago, when reading a story written by Dr. William A. Hammond, entitled "Lal," we came across a dissertation relating to breakfast eating as applied to women, that possesses much common sense and which we here quote in detail:

> Perhaps there is no better test of a woman's health and beauty, than her appearance when she presents herself at an early breakfast table. She is then more as Nature made her than at any other period of the day, when art has been brought in with a view of heightening her charms. If she has slept well, it argues, to some extent, a sound nervous system, and the effect is seen in the brightness of her eyes and the tone possessed by the muscles of the face and neck. Her movements are full of grace, for her limbs have been refreshed and strengthened by repose, and her mind is clear and bright, for it also has rested, and there have been no bad dreams to exhaust her nervous system and make her limp and haggard. Her intelligence is then at its maximum, and she feels the mental recklessness that is so generally the result of sound, healthy sleep, and that is only a natural elation of the emotions; pleasant, doubtless, for her to exhibit, but far more pleasant to those to whom it is manifested. If, on the contrary, she has slept badly, or has suffered from nightmare in consequence of a feeble digestive system, her eyes are weak, dim, and watery, her face is flabby, her head appears to be held unsteadily on her shoulders, for it droops on her chest, or bobs helplessly from side to side, her complexion is dull and blotchy, red where it ought not to be red, and pale where it ought not to be pale. Her expression is indicative of the discomfort she has undergone during the night, her movements are either painfully slow or aggravatingly brusque, her intellect shows stupidity, her emotions are torpid, her perception dull.

While the woman that is in good physical health exhibits all the beauty in the early morning that her features are capable of expressing, the one whose organic life is deranged is at this period of the day at her worst. There is no better test of a woman's health than her ability to eat a hearty breakfast, and it might also be said that her physical beauty is in direct proportion to the amount of beefsteak or mutton chops she can put into herself at this meal. Certainly, pretty women can always eat a hearty breakfast."

We could wish that every intelligent woman might read the foregoing, which contains at once the ethics and law of good health applicable to the female sex. Women are not only prone to light eating at the morning meal but also are much given to the ingestion of the wrong food. It is quite important that adequate nourishment should be taken reasonably early in the morning. The "coffee and roll" habit is to be deprecated. The body stands in greater need of a substantial morning meal than at any other time during the day; the longest fast occurs between dinner and breakfast; the stomach has remained empty for a number of hours, hence has become rested and ready to receive and digest food. Therefore, adequate nutritious food elements should be taken in the morning.

On the other hand, a hearty luncheon is a mistake; it makes one dull during that portion of the day when the mentality should be most active, and when prolonged into the middle of the afternoon leaves the stomach unprepared for a hearty dinner. After an active day the society woman can enjoy a good dinner, and the business man needs it. But each of these important groups of community should by their methods and habits from breakfast to dinner, or in other words from early morning until the shades of evening, so conduct the affairs of life as to relish, ingest and digest a wholesome dinner.

More power to the young student of Trinity College, Hartford, who spent his Easter vacation studying tramp life as a tramp. If after he has reached home and had a bath he still recognises himself and retains his impressions of the road, he may find that he has learned something of value and, if he will give a bond with two good sureties not to communicate his impressions to a 10-cent magazine and pose as a Socialist, all may be forgiven him.

There are worse avocations than tramping for a student with ambition to improve himself. Among these I should place waiting at table, blacking boots or doing the like menial services at a summer hotel. I know that use has set the seal of approbation on these latter employments as aids to impecunious young men of decent birth ambitious of getting an education. At the resort where I spent a part of last summer, my coffee was handed to me by a budding bachelor of arts and my boots were blacked by a student in medicine. These persons took my tips without shame and were not ashamed to render very indifferent service in return therefor. If the bachelor is no better at his books than at waiting at table he is but a dull scholar, and if the medical student cannot learn to black boots I should despair of his ability to become a bone-setter.

The twain, in a word, were frauds and an imposition. They confirmed me in the opinion I have held for many years, that if a young man has the makings of a good menial servant he is not fit to be graduated from college, and if a student take pay for services he can't render, he is obtaining money under false pretenses. The student waiters are welcome to take either end of this, as pleases them.

The foregoing, clipped from the New York Morning Telegraph, April 23, 1908, contains a grain of suggestion that may well claim the attention of undergraduates in general, and those of medicine in particular. Whatever is worth doing at all is worth doing well. This axiomatic truth often has been stated, sometimes in one way and again in another, but always with the same import. A young student of Columbia University lost his life yesterday (May 20, 1908), because of a foolish prank. He was shot while attempting to get away from a policeman, who caught two students breaking into a small candy store. Such pranks do not contribute to the dignity of character that should pervade the entire fabric of educational life. We hope the lesson contained in the foregoing clipping, as well as the one pertaining to the sad death of the Columbia student, will be heeded by those to whom they may apply, if any such may chance to read this paragraph.

---

It is reported that, in a damage suit brought by a Harvard graduate (*New York Tribune*), against an express company for the loss of his medical diploma, President Eliot and Harvard professors testified that "a diploma has only a sentimental value." This deposition apparently explains why students are allowed to earn diplomas by pursuing studies of sentimental value. Economically a college diploma is thus made a *quid pro quo* "in kind" for courses in Mesopotamian Mezzotints and the Esoteric Mental Calisthenics of Hegelianism.

---

Governor Hughes recently signed Senator Cassidy's bill, which declares tuberculosis to be an infectious disease and provides for the reporting of all cases to the local health authorities. It also provides for the free examination of sputum by the health authorities and for the disinfection of the premises after death or removal of a person having tuberculosis.

---

The Board of Supervisors offered to sell to the University of Buffalo, for $50,000, 100 acres of the almshouse farm, reserving the tract where the present almshouse stands, as well as the site of the county hospital. Yesterday the University council submitted a counter proposition, asking for five additional acres, including a portion of the land now occupied by the Almshouse building. It proposes, however, to give the county an easement on the land occupied by the almshouse building for such time as it shall be used for purposes of an almshouse. The council further offers to pay $5,000 in cash and the remainder in one year, and it will consent to a stipulation that, if a university is not built on the site within ten years, the county may buy the land back at cost and interest.

The difference between the supervisors and the council is very slight. The county would retain the privilege of maintaining the old almshouse on the site as long as it wishes. That was the only purpose of reserving the portion of the farm which the building covers. As a matter of fact, the old building ought to be moved away as quickly as possible, whether the University ever acquires the site or not. The council has made a more liberal offer than many friends of the University thought should be asked of it, considering the public purpose to which the land is to be put. It is hoped the supervisors will consent to the terms.

---

The Commissioner of Health, of Buffalo, Dr. Ernest Wende, recently stated that the health department will open a food and drug laboratory July, 1908. A microscopist and chemist will be in charge. Samples of all food, sold in the city will be examined there.

Governor Hughes has approved the bill of Senator Wilcox providing for the regulation of the practice of optometry, and vetoed two measures of general interest. One was designed to make October 12, Columbus Day, a legal holiday, and the other was intended to provide free transportation to letter-carriers, policemen and firemen, "while in uniform."

